# Temporal and Spatial Epidemiological Analysis of Peste Des Petits Ruminants Outbreaks from the Past 25 Years in Sheep and Goats and Its Control in India

**DOI:** 10.3390/v13030480

**Published:** 2021-03-15

**Authors:** Vinayagamurthy Balamurugan, Kirubakaran Vinod Kumar, Ramesh Dheeraj, Rashmi Kurli, Kuralayanapalya Puttahonnappa Suresh, GurrappaNaidu Govindaraj, Bibek Ranjan Shome, Parimal Roy

**Affiliations:** Indian Council of Agricultural Research -National Institute of Veterinary Epidemiology and Disease Informatics (ICAR-NIVEDI), Post Box No. 6450, Yelahanka, Bengaluru 560 064, Karnataka, India; vinodkmr33@gmail.com (K.V.K.); ddheerajr229@gmail.com (R.D.); rashmikurli@yahoo.in (R.K.); sureshkp97@rediffmail.com (K.P.S.); mggraj74@gmail.com (G.G.); brshome@gmail.com (B.R.S.); parimalroy580@gmail.com (P.R.)

**Keywords:** PPR, epidemiology, 1995–2019, temporal-spatial analysis, risk zones, endemicity, control, India

## Abstract

This study was aimed to understand the temporal and spatial epidemiology of peste des petits ruminants (PPR) in India using national surveillance data available in the National Animal Diseases Referral Expert System (NADRES) along with its control plan undertaken. On analysis of the outbreaks/cases reports in sheep and goats in NADRES database from 1995 to 2019, it was observed that PPR features among the top ten diseases and stands first among viral diseases, and among reported deaths, PPR accounts for 36% of mortality in sheep and goats. PPR outbreaks occur round the year in all the seasons but are encountered most frequently during the lean period especially, in the winter season (January to February) in different regions/zones. The reported outbreaks have been progressively declined in most of the states in India due to the implementation of a mass vaccination strategic program since 2011. On state-wise analysis, the PPR risk-areas showed wide variations with different levels of endemicity. Andhra Pradesh, West Bengal, and Karnataka were the top three outbreaks reported states during 1995–2010, whereas Jharkhand and West Bengal states reported more outbreaks during 2011–2015 and 2016–2019 periods. The temporal and spatial distribution of PPR in India provides valuable information on the hotspot areas/zones to take appropriate policy decisions towards its prevention and control in different regions/zones of India. The study also identifies when and where intensive surveillance and vaccination along with biosecurity measures need to be implemented for the control and eradication of the disease from India in consonance with the PPR Global Control and Eradication Strategy.

## 1. Introduction

Peste des petits ruminants (PPR), otherwise known as ‘small ruminants plague’, is one of the highly contagious, World Organisation for Animal Health (OIE) notifiable, economically important transboundary devastating viral diseases of sheep and goats. The disease is associated with high morbidity and mortality and is caused by the *Small ruminant morbillivirus* (otherwise known as PPR virus-PPRV) [1]. Clinically, the disease is manifested by high fever, discharges from eyes and nasal orifices, necrotizing oral lesions, erosive stomatitis, enteritis, diarrhea, and bronchopneumonia followed by either death of the animal or recovery from the disease [2]. PPR was first reported in the Côte d’Ivoire, West Africa, and later from other parts of the world viz. the parts of Africa, the Middle East, the parts of Asia, and the parts of Europe. Recently, outbreaks of PPR are being reported from several new countries in Africa and Asia, and at present more than 70 countries have confirmed PPR affecting ~1.7 billion of the global sheep and goat population [3]. The spread of disease to several new countries in Africa, Asia, and Europe with the involvement of various lineage of PPRV is a cause of global animal health concern especially the recent introduction of Asian lineage in some African countries and the introduction of PPR in Southern Europe through Turkey [4,5,6]. Because of the huge impact on production, PPR is considered one of the main constraints in augmenting productivity in small ruminants in endemic countries. Considering the importance of small ruminants in ensuring food security and socio-economic growth in many parts of the world, mainly in Africa and Asia, a global consensus was agreed on the need to eradicate PPR with the adoption of the PPR Global Control and Eradication Strategy (GCES) with a vision to make the world free from PPR by 2030. In this direction, FAO and OIE, launched the PPR global eradication program (PPR-GEP) for the period 2017–2021 with the adoption of PPR GCES for global elimination of PPRV by 2030 [7]. 

India is a vast country with a population of ~148.88 million goats and 74.26 million sheep (as per the 20th Livestock census-2019) [8], as against approximately 2.1 billion world population of sheep and goats. In India, PPR was first reported from Tamil Nadu state in 1987 [9]. The disease was believed to be restricted in Southern India until severe epidemics swept through the rest of India in 1994 [10]. Since then, the disease became endemic in many states of India [2]. Despite strict control measures including statutory regulations along with the availability of vaccines and diagnostics, this infection remains a constant threat [11]. PPR is a major obstacle in augmenting productivity [12] and adversely affects the livelihood of marginal and small farmers as well as landless laborers, as sheep and goats are an important productive asset and generate income and employment throughout the year for these farmers. The Department of Animal Husbandry and Dairying (DAHD), Government of India (GoI) implemented a national PPR-Control Program (PPR-CP) even before PPR GCES, during 2010–2011 [13] to control and eradicate PPR from India in a time-bound manner on the lines of rinderpest (RP) eradication [14]. In the first phase of PPR-CP, the states and Union Territories (UTs) in Southern India were included in the vaccination, and the remaining states and UTs of India were included in the second phase during 2014–2015 [14]. The activities implemented under PPR-CP includes vaccination, pre-, and post-vaccination monitoring, attaining PPR free zone with vaccination, clinical surveillance, outbreaks investigation, response, and communication [14], and implementation of focused ring vaccination in place with biosecurity precautionary measures for prevention and spread of outbreaks or to minimize the risk of spread including containments of the infectivity, severity, and transmissibility into an animal population. In India, several outbreaks of PPR in sheep and goats have not been recorded properly, owing to inadequate animal disease surveillance and reporting systems [15,16]. Further, a national-wide serosurvey conducted by employing the PPR competitive ELISA [17] in sheep and goats during 2017–2018 showed a wide variation in the prevalence status of PPRV antibodies (seroprevalence/immune population) in the different states of India as vaccination strategies employed and coverage by the states varied significantly [18,19,20,21], even though it is not possible to distinguish the vaccinated and infected animals, as the differentiation of infected from vaccinated animals (DIVA) vaccine was not being used in the PPR-CP. 

Epidemiological analysis of outbreaks/cases [An outbreak is defined as more cases or a sudden increase in occurrences of an infectious disease (epidemics) in a particular time and place than expected; cases refers to the number of animals that become ill with (or die from) a disease in a population initially free of the disease during an outbreak] data from different geographical areas with varying agro-climatic conditions may help in devising effective vaccination and control strategies, to prevent the disease incursion, and to acquire disease-free status by implementing the effective comprehensive active surveillance and intensive monitoring program. This would help in sustainable livestock production and management for the livelihood of farmers and paves a way for increasing small ruminant exports due to disease-free status [20]. So far, no such nationwide studies have been conducted to understand the temporal variations and spatial distribution of the PPR in India. Further, it is imperative to understand disease dynamics, disease hotspot areas/zones, and time of outbreaks to support policymakers to take appropriate decisions towards the control and eradication. Therefore, the present study was undertaken to identify the temporal pattern and spatial distribution of PPR outbreaks in sheep and goats from 1995 to 2019 using available nation-wide passive surveillance data (outbreaks/cases), as well as to understand the status and effect of vaccination carried out under ongoing PPR control program in India. 

## 2. Materials and Methods

### 2.1. Study Area Description 

**Figure 1:**
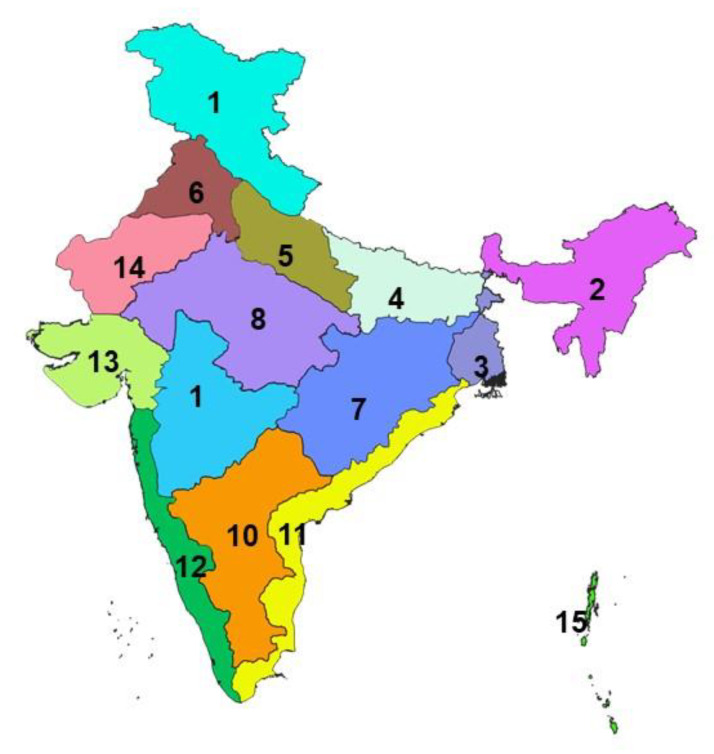
Shows different agro-climatic zones of India. 1.Western Himalayan 2. Eastern Himalayan 3. Lower Gangetic Plains 4. Middle Gangetic Plains 5. Upper Gangetic Plains 6. Trans Gangetic Plains 7. Eastern Plateau & Hills 8. Central Plateau & Hills 9. Western Plateau & Hills 10. Southern Plateau & Hills 11. East Coast Plains & Hills 12. West Coast Plains & Hills 13. Gujarat Plans and Hills 14. Western Dry Region and 15. Islands.

The livestock sector contributes 25.6% of total Agriculture Gross Domestic Products (GDP) and 4.11% national GDP and 16% to the income of small farm households as against an average of 14% for all rural households and employs about 8.8% of the population in India [22]. The small ruminant sector is a strong contributor to the income and livelihoods of the poorest segments of the society, and provides sustenance to the rural population, and contributes significantly to poverty alleviation [23]. In India, the climate differs significantly from one region to another across the length breadth of the country, resulting in significant variations in the distribution of agro-climatic livestock production systems. There are different agro-climatic zones of India [24], covering 28 states and 8 UTs in the different geographical regions viz., Sothern plateau and hills, and West coastal plateau and hills zones covering southern parts (South zone); Eastern Himalayan zone covering north-eastern states (North-East zone), Central and Western plateau and hills and Western Dry as well as Gujarat plains and hills zones covering the central and western India (Central and Western zone), Western Himalayan zone and Upper and Trans Gangetic Plains zones covering northern states (North zone), and the East coast plains and hills and Middle Gangetic Plains and Western plateau and hills zones covering Eastern parts (East zone). The different agro-climatic zones of the country [24] are depicted and shown in the Indian map (Figure 1).

### 2.2. Data Source 

The national surveillance epidemiological data on PPR in India from 1995–2019 were obtained from the National Animal Diseases Referral Expert System (NADRES) database of the Indian Council of Agricultural Research-National Institute of Veterinary Epidemiology and Disease Informatics (ICAR-NIVEDI). Every month the epidemiological parameters of various diseases including PPR are being collected from AICRP (All India Coordinated Research Project) on Animal Disease Monitoring and Surveillance (ADMAS) collaborating centers of ICAR- NIVEDI located in various States and UTs, and compiled at ICAR-NIVEDI and maintained as the NADRES database at ICAR-NIVEDI [25]. The outbreaks data includes the number of outbreaks, risk populations, attacks, affected species (sheep or goats and goats/sheep), and outbreak locations (block/district and state). In this study, each of the outbreak(s) represents the disease that occurred in an area, which is pooled at district levels in the state to get the total number of outbreaks in a given period. Similarly, attacks represent the number of animals affected by disease ie., cases in each of the outbreaks, which is pooled at district levels in the state to get the total number of cases in a given period. In the disease database, some outbreaks have been presented as sheep and goats together without clear demarcation of species, hence, it was considered together in the analysis. The disease data were collected via passive surveillance every month by the Assistant Director of the block (s) of the particular district in animal husbandry departments, through local field veterinarians of the Veterinary Dispensary. Generally, the farmers voluntarily report to the local field veterinarian, if their animals become sick. Among reported, some of the outbreaks have been diagnosed based on clinical signs only by professional field veterinarians while most of the outbreaks were confirmed by designated district/state/national animal disease diagnostic laboratories. Further, epidemiological data on PPR and available vaccination details {The vaccination data for the population of small ruminants were available for the financial year (FY) [i.e., the period between 1st April and 31st March of the next year (12 months) is considered as one FY] were obtained from the majority of the state animal husbandry departments for some of the studied years and used for epidemiological analysis and to assess the effect of vaccination.

### 2.3. Data Analysis

The passive disease surveillance data available at the NADRES database was analyzed in Microsoft Excel 2016 (Microsoft Corporation, Redmond, WA, USA) and were grouped into three periods as, before the implementation of the national PPR-CP (1995–2010), after implementation of the first phase (2011–2015), and the second phase of PPR-CP (2016–2019). Further, passive surveillance data were collated at the regional level, and cumulative monthly outbreaks/cases of PPR were calculated for identifying the seasonal occurrence of the disease in different zones. Based on the cumulative reports, the status of the disease in sheep and goats, disease burden, regional distribution, host susceptibility, the decadal, quinquennial, and yearly temporal and seasonal patterns, spatial distribution, risk zones/areas, endemicity, etc. in the different zones and states of India has been analyzed. All maps were generated using the open-source GIS software QGIS (Quantum GIS Development Team 2018, QGIS version 2.18.0, QGIS Usergroup, Switzerland) to visualize risk zones/ areas and outbreak locations of the districts in the different states of India. The categorization of endemicity was based on the scale of the cumulative outbreaks that occurred in the area (district) per year in the given period of analysis and classified into different categories of risk (if the outbreaks numbers as 0- no risk, 1- very low, 2- low, 3- medium, 4- high and >4 very high-risk districts) and are depicted in the map. Further, this categorization is also classified as sporadic (very low = 1 outbreak), low endemic [mild = 2 outbreaks and moderate = 3 outbreaks)}, highly endemic (high = 4 outbreaks), and hyperendemic (very high ≥ 4 outbreaks] districts, respectively to present the different endemicity levels in the Indian states. The collected outbreaks and vaccination data up to FY 2019–2020 were analyzed to assess the effect of vaccination. 

Linear trend analysis using the regression method was performed to understand the temporal pattern of outbreaks and cases. The mean/median cases per outbreak and range and standard deviation (SD) of the cases were calculated to understand the variabilities of the cases during different outbreaks. To elicit which variables are likely to be associated with the number of outbreaks, the stepwise multivariable linear regression method was fitted for sheep, goats, and pooled data with the number of outbreaks as the dependent variable, and zones, years, and seasonal dummies as the independent (explanatory) variables in R software (R version 4.0.3, UseR! 2020, St. Louis, Missouri, USA). The month was not considered as an independent variable separately, since the disease cycle is mostly seasonal, therefore, months have been converted into six seasons Viz. Winter (January–February); Spring (March–April); Summer (May–June); Monsoon (July–August); Autumn (September–October) and Pre-winter (November- December) based on the outbreaks months. The reference group for the dummy explanatory variables for seasons, zones and years, is the Autumn season, Central zone, and the year 1995, respectively. Further, the proportion of outbreaks and cases to the small ruminant population in respective states were calculated using the 17th (2003), 18th (2007) and 19th (2012) and 20th (2019) Livestock census [8] for the periods 1995–2003, 2003–2010, 201–2015, and 2016–2019, respectively.

## 3. Results

### 3.1. Disease Burden 

**Figure 2:**
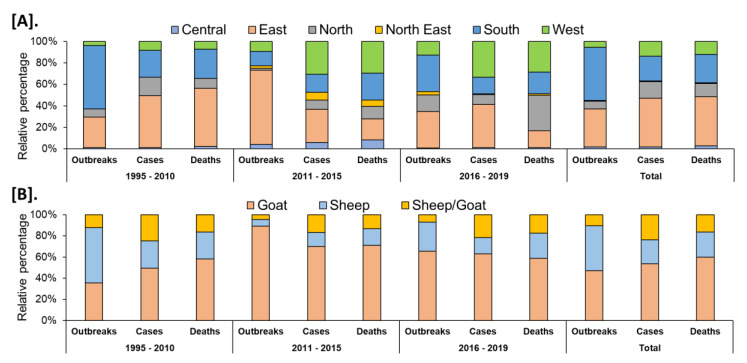
Analysis of cumulative PPR reports in different periods (1995–2019). (**A**). Zone-wise (**B**). Species-wise. Relative percentages of the cumulative outbreaks, cases, and deaths in sheep and goats in different zones of the country during the different analyzed periods. The highest proportion of reported outbreaks and deaths was observed in the South and East zone, respectively.

**Figure 3:**
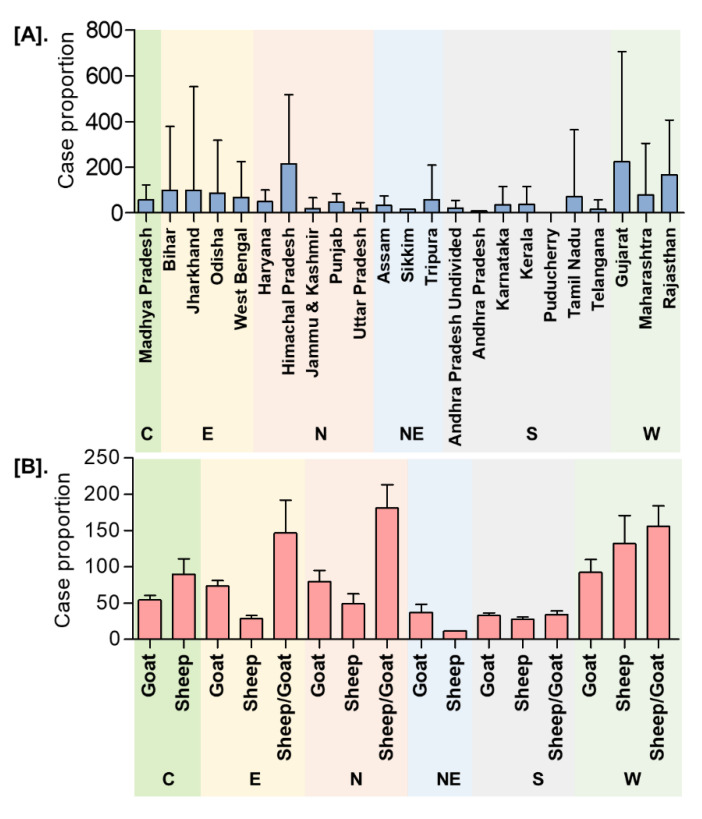
Dispersion analysis of the cumulative PPR reports in India (1995–2019). (**A**). State/Zone-wise (**B**). Species-wise. The mean cases per outbreak with the measure of the variation was calculated. C-Central zone; E-East zone; N- North Zone; NE-North-East Zone; S-South Zone; W-West zone. The highest mean cases per outbreak were observed in Gujarat, followed by Himachal Pradesh, Rajasthan, with the highest in sheep and goats in the West and North zone, respectively.

**Figure 4:**
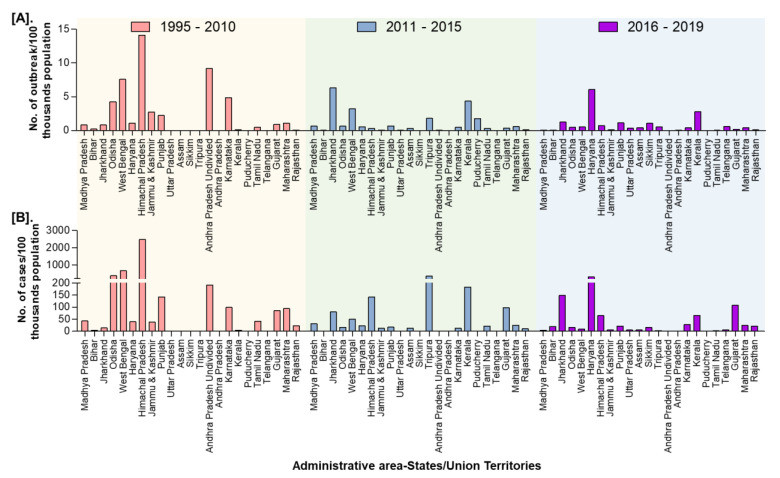
State-wise proportion of the cumulative PPR reports in India (1995–2019) with the population. (**A**). Outbreaks (**B**). Cases. Himachal Pradesh, West Bengal, and Odisha were the top three states reporting the highest number of cases per 100 thousand population during 1995–2010, whereas Tripura & Kerala and Jharkhand & Haryana states were highest during 2011-15 and 2016–2019 periods, respectively.

In India, a total of 8168 outbreaks of PPR were reported from 1995 to 2019 with the highest 3844 outbreaks in goats followed by 3473 outbreaks in sheep and 851 outbreaks in the flocks where sheep and goats are reared together. Among different geographical regions, the South zone reported the highest proportion (4029 outbreaks; 49.33%) of reported outbreaks followed by the East (2896 outbreaks; 35.46%), North (600 outbreaks; 7.35%), and West (449 outbreaks; 5.50%) zones, whereas the Central (140 outbreaks; 1.71%) and North-East (54 outbreaks; 0.67%) zones reported fewer outbreaks. Further, it has been observed that PPR features among the top ten diseases (PPR, sheep & goat pox, Rabies, Enterotoxaemia, Bluetongue, Coccidiosis, Babesiosis, Theileriosis, Footrot, and Fascioliasis) in sheep and goats and stands first among viral diseases, and accounts for 36% of the mortalities among reported deaths based on the analysis of the outbreaks/cases reports in NADRES database from 1995 to 2019. On analysis of 25 years data, it was observed that 95,492 deaths were reported, of which 59.76% in goats (n = 57,066), 23.98% in sheep (n = 22,901) and 16.26% in sheep and goats together (n = 15,525). Further, on comparison, the East zone (45.69%) showed the highest proportion of reported deaths followed by the South (26.29%), West (12.23%), North (12.12%), Central (2.84%), and North-East (0.83%) zones. The cumulative deaths reported during 1995–2010 was highest (76.89%; n = 73,428) followed by 11.82% (n = 11,286) during 2011–2015 and 11.29% (n = 10,778), during 2016–2019 and 2.86% (n = 2729) deaths only in 2019 were reported. The state-wise detail of case fatality rate (CFR) of PPR reports in sheep and goats in different zones is presented in Figure S1. The details of the PPR outbreaks and cases in sheep and goats in different zones in different periods of analysis are presented in Figure 2. Further, the mean cases per outbreak with the measure of the variation like range and SD were calculated statistically to understand the variabilities of the cases during different outbreaks and the results are summarized and presented in Figure 3. Furthermore, the results of outbreaks and cases in proportion to the population for different periods are presented in Figure 4. Himachal Pradesh (HP), undivided Andhra Pradesh (AP), and West Bengal (WB) states were the top three states reporting the highest number of outbreaks per 100 thousand population during 1995–2010, whereas Jharkhand and Haryana states were highest during 2011–2015 and 2016–2019 periods, respectively. The proportion of reported cases to the population was highest in HP, WB, Odisha states in 1995–2010, Tripura & Kerala states in 2011–2015, and Jharkhand and Haryana in 2016–2019.

### 3.2. Temporal Patterns

**Figure 5:**
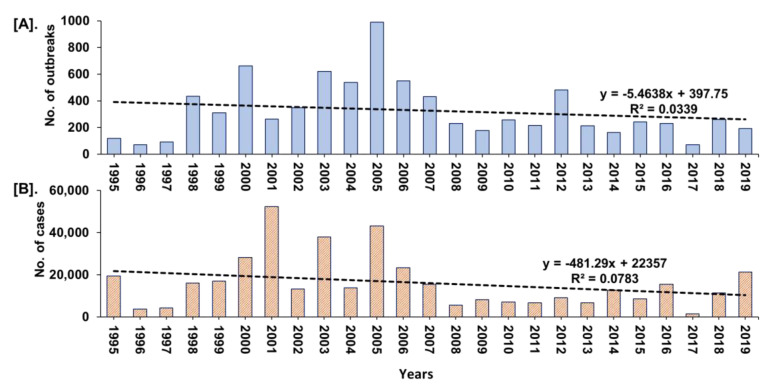
Year-wise trend analysis of cumulative PPR reports in India (1995–2019). (**A**). Outbreaks (**B**). Cases. A gradual increase in outbreaks and cases since 1995 and the highest numbers between 2000 and 2007, with a declining trend from 2005 was observed.

The year-wise trend analysis of cumulative PPR reports in India (1995–2019) is shown in Figure 5. The cumulative PPR outbreak report showed a gradual increase in outbreaks and cases since 1995 and the highest numbers were reported between 2000 and 2007 with a declining trend from 2005. However, during 2018 and 2019, the marginal increase in the number of outbreaks (Figure 5) in defined geographical areas in Maharashtra, Haryana, Jharkhand, and West Bengal when compared to 2017 was observed.

**Figure 6:**
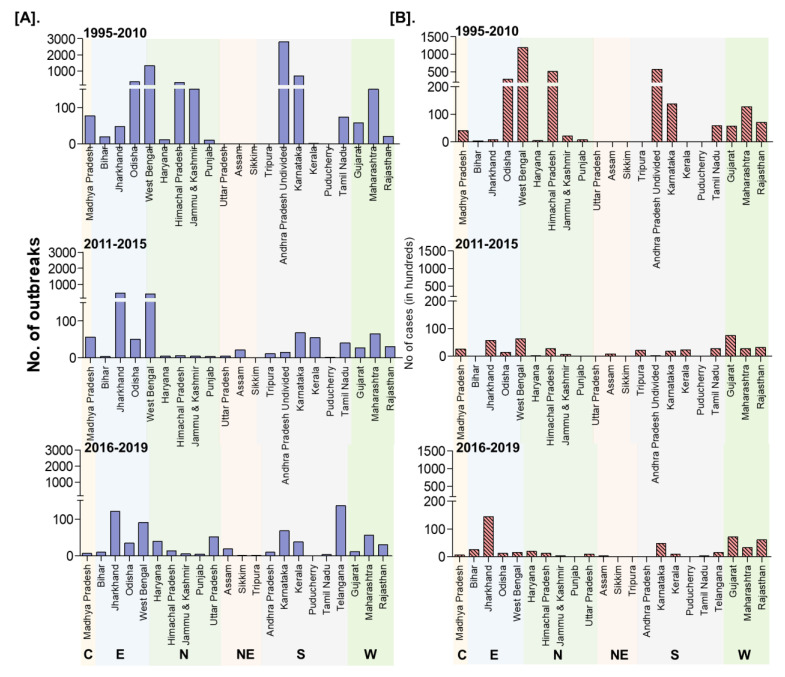
The state-wise occurrence of cumulative PPR reports in India (1995–2019). (**A**). Outbreaks (**B**). Cases. C-Central zone; E-East zone; N- North Zone; NE-North-East Zone; S-South Zone; W-West zone. Karnataka and Andhra Pradesh have shown a decline in the number of outbreaks during 2011-FS115 and 2016-19 with sporadic outbreaks in 2018 and 2019; West Bengal and Jharkhand states have reported the highest outbreaks during 2011–2015 and 2016–2019 periods, respectively.

Further, the zone-wise analysis revealed that East zone reported highest cases (181,824 cases; 45.17%) followed by the South (92,018 cases; 22.86%); North (62,288 cases; 15.48%); West (55,701 cases; 13.85%) zones, whereas the Central (7252 cases; 1.80%) and North-East (3,412 cases; 0.85%) zones reported the lowest number of cases (Figure 6). Furthermore, the highest (74.62%) proportion of reported outbreaks (n = 6095) were during 1995–2010 followed by 16.12% (n = 1317) during 2011–2015 and 9.26% (n = 756) during 2016–2019. Nevertheless, the reported outbreaks during 1995–2010, ranged from 2 to 2,807 with least in Kerala and highest in Andhra Pradesh with an average of ~380 outbreaks, whereas 1 to 451 outbreaks were reported during 2011–2015, with least in Puducherry and highest in Jharkhand, and 1 to 136 outbreaks with least in Sikkim and highest in Telangana (n = 136) followed by 121 outbreaks in Jharkhand during 2016–2019.

**Figure 7:**
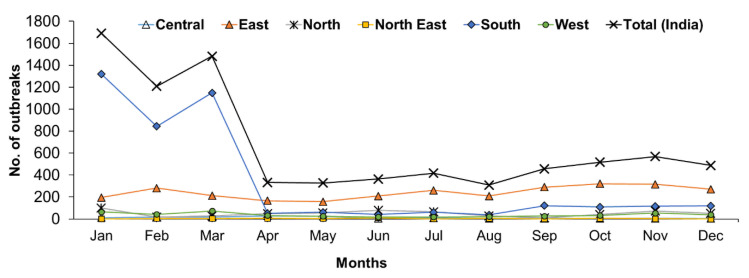
Month-wise analysis of cumulative PPR outbreaks in different zones in India (1995–2019). The outbreaks were predominantly observed between December to April in the South zone, during October & November in the East zone; April & May months in the Central zone; in June in the North-East zone; from January to March in the Western zone and between November and January in the North Zone.

The month-wise reports indicated that PPR has been found to occur throughout the year and more outbreaks occurred from January to March (Figure 7). The monthly analysis also revealed that outbreaks were predominantly observed between January to March in the South zone (Figure 7), whereas outbreaks were reported during October & November in the East zone and April & May months in the Central zone. In the North-East zone, the highest outbreaks were observed in June months only. In the Western zone, most of the outbreaks were reported from January to March followed by November month, whereas in the North Zone, the highest outbreaks were reported between November and January followed by June and July. Further, on comparison, more cases were recorded during June and July months in the East and North zone, respectively, whereas in the West, Central and North-East zones, during November, May, and June months and in the south zone more cases were recorded during December to April months. 

On analysis of multivariable linear regression coefficients, the results (Table S1A) indicated that, a significant increase of 18 more PPR outbreaks in sheep in the South zone as compared to the Central zone (reference zone), accompanied by a significant increase in the number of outbreaks in the Winter (16 more outbreaks) and Spring seasons (seven more outbreaks), compared to the Autumn season (reference season). Further, there was a significant increase in the number of outbreaks during 2000 and 2005, followed by 2004, which was 17, 18, and 12 more outbreaks compared to the year 1995(reference year). In Goats, the results (Table S1B), indicated that, a significant increase of 17 and 3 more PPR outbreaks in the East and South zones as compared to the Central zone, accompanied by a significant increase of 11, 9, and 5 more outbreaks during 2012, 2005, and 2006 & 2007, respectively as compared to the year 1995. The pooled analysis for sheep and goats outbreaks (Table S1C) revealed that a significant increase of 26 and 18 more outbreaks in sheep in South and East zones as compared to the Central zone, accompanied by a significant increase in the number of outbreaks in the Winter season, compared to the Autumn season. Further, a significant increase in the number of outbreaks by 24, 15, 14, 12, and 11 during 2005, 2000, 2006, 2003, and 2004, respectively, as compared to the year 1995. The details of the estimated multivariable linear regression coefficients of important parameters associated with PPR outbreaks in sheep and goats are presented in Table S1.

### 3.3. Species Susceptibility

**Figure 8:**
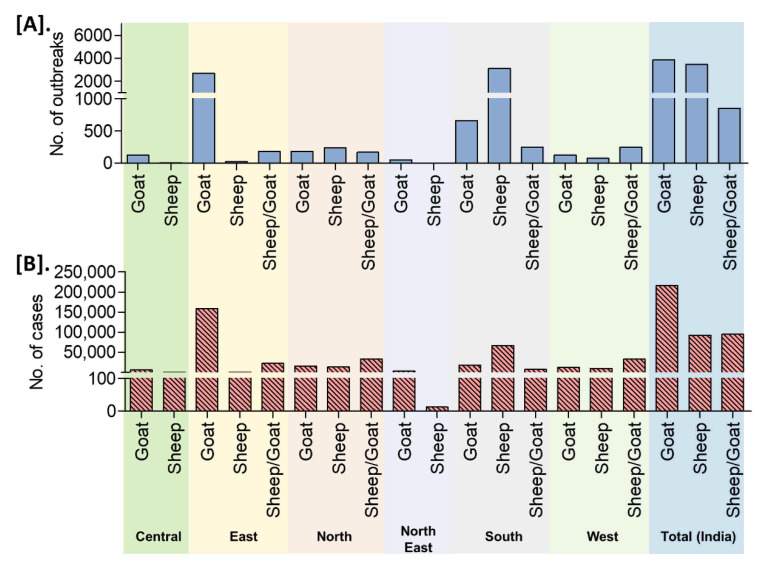
Species-wise occurrence of cumulative PPR reports in different zones/regions of India (1995–2019). (**A**). Outbreaks (**B**). Cases. An increased number of outbreaks have been reported in goats than in sheep in the different zones of the country, except in the south zone where the number of outbreaks was greater in sheep.

On analysis of the reported outbreaks and cases (outbreaks n = 8168, cases n = 402,495) from 25 years, the highest outbreaks (47.06%) in goats followed by 42.52% in sheep and 10.42% in sheep and goats together with 53.52% (n = 215,425), 22.91% (n = 92,202), and 23.57% (n = 94868) cases in goats, sheep, and sheep and goats together, respectively. The detailed species-wise reported PPR cases/outbreaks are presented in Figure 8. An increased number of outbreaks have been reported in goats than in sheep in the different zones of the country, except in the south zone where the number of outbreaks was higher in sheep (Figure 8). 

### 3.4. Spatial Distribution

**Figure 9:**
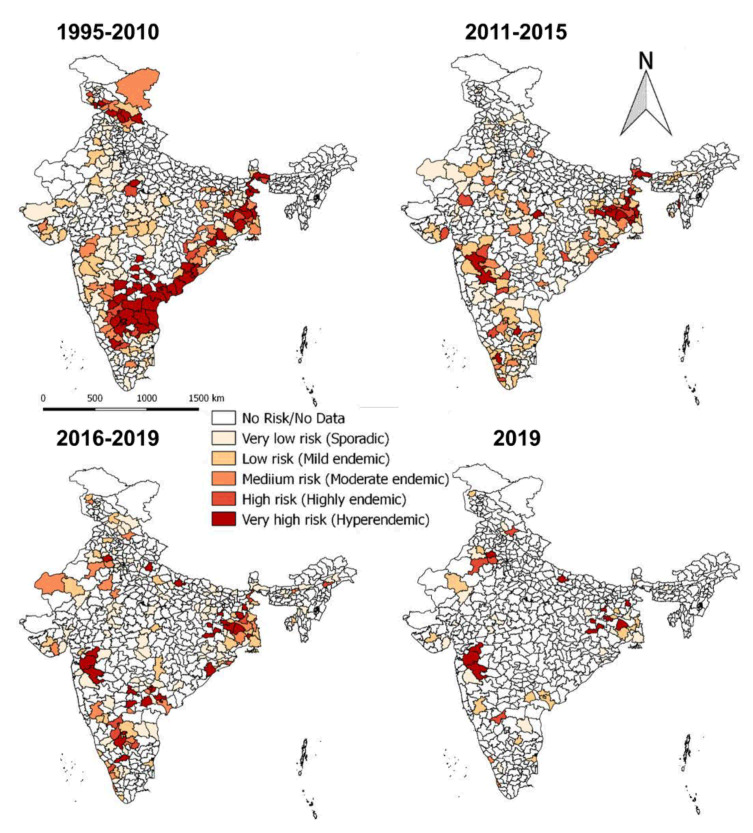
Endemic districts of PPR in different states of India (1995–2019). The endemicity categorization is based on the scale of the cumulative outbreaks, that occurred in the districts per year in the given period of analysis and classified as in the categories of risk, if the outbreaks numbers in the district as 0 –no risk, 1- very low, 2- low, 3- medium, 4- high and >4 very high-risk districts depicted in the map. Andhra Pradesh, West Bengal, and Karnataka states were the top three states during 1995–2010, whereas during 2011-15 and 2016–2019, Jharkhand and West Bengal states had reported more PPR outbreaks. The current status of PPR outbreaks during 2019 in different districts of India was depicted separately in the map.

On analysis of outbreaks data, PPR endemic risk areas showed a wide variation in the different states/zones of India at different periods. Based on the occurrence of the cumulative outbreaks in the endemic districts per year in the given period of analysis, the districts in four different categories in different states of India were obtained as sporadic (1 outbreak), low endemic (2–3 outbreaks), highly endemic (4 outbreaks), and hyperendemic (> 4 outbreaks) districts, respectively and are summarised in Table S2. Furthermore, the different categories of endemic districts are depicted in six scale classification at different periods of analysis and are depicted in the map (Figure 9) to visualize risk zones/areas and the number of outbreaks that occurred in district locations in different states of India. Many districts of Andhra Pradesh, Karnataka, West Bengal, Jharkhand, Tamil Nadu, Maharashtra, Gujarat, Himachal Pradesh, Jammu and Kashmir, and Odisha fall under the highly endemic and hyperendemic risk areas categories, whereas many districts in the states of Central and North-East zones belongs to sporadic and low endemic risk areas. An increasing number of districts were affected with PPRV infection during 1995–2010 compared to the 2011–2015 and 2016–2019 periods (Figure 9). Further, on analysis of cumulative data, Andhra Pradesh, West Bengal, and Karnataka states were the top three states during 1995–2010, whereas, during 2011-15 Jharkhand, West Bengal and Karnataka, and during 2016–2019, Jharkhand, West Bengal, and Maharashtra & Karnataka had reported the highest number of outbreaks (Figure 9). Furthermore, East and South zones had reported more outbreaks than other zones during 1995–2010, whereas during 2011-15 and 2016-19 more outbreaks were observed only in the East zone. States like Jharkhand, West Bengal, Kerala, and Rajasthan have reported an increasing trend of PPR outbreaks during 2011–2015. 

Although South zone states like Karnataka, Andhra Pradesh have shown a decline in the number of outbreaks and the disease is still reported sporadically. As of now (in 2019), PPR is being restricted to only a few districts in the mass vaccinated program regularly implemented states (Karnataka/Andhra Pradesh) and more numbers of districts in the non-vaccinated or focused vaccination practiced states (Jharkhand, Maharashtra, Uttar Pradesh, Haryana, West Bengal, Rajasthan, Kerala, etc.,) of India.

### 3.5. Effect of Vaccination 

**Figure 10:**
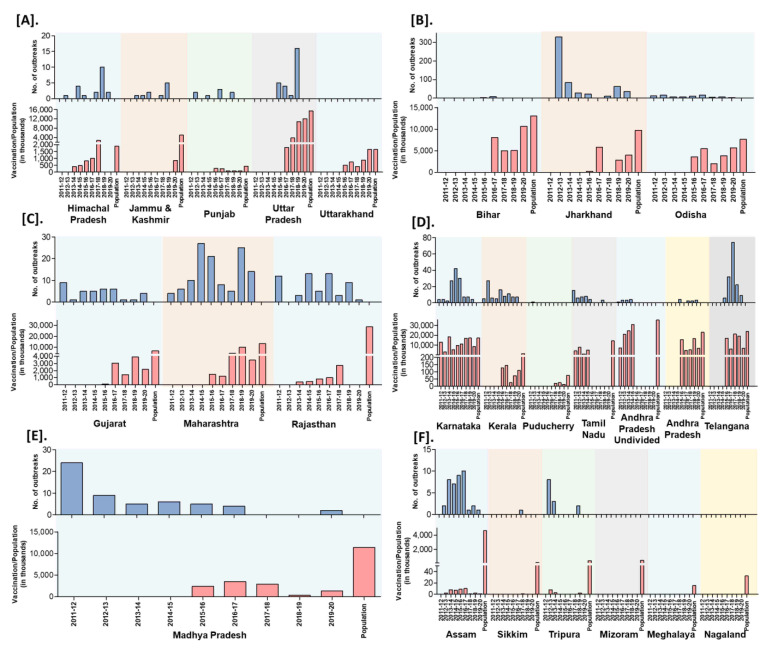
Zone-wise trend of outbreaks reports in the states from the financial year 2011-12 to 2019–2020; outbreak reports versus the doses of vaccines used (in thousand) in the strategic immunizations during the preceding years against the population are depicted. (**A**). North (**B**). East (**C**). West (**D**). South (**E**). Central (**F**). North-East zones.

India practiced focused vaccination (vaccination limited to the place of the outbreak with the radius of 3–10 km to contain the disease spread) during outbreaks to control and prevent the disease spread in 15 states (AP, Karnataka, Himachal Pradesh, Uttar Pradesh, Madhya Pradesh, Uttarakhand, Chhattisgarh, Haryana, Jammu and Kashmir, Jharkhand, Maharashtra, Odisha, Punjab, Rajasthan, and West Bengal) of India since 2002 under the respective state’s sponsored or Assistance to States for Control of Animal Diseases (ASCAD) program of the GoI. Further, besides immunization of susceptible animals, implementation of biosecurity measures during outbreaks has been carried out by the State Government to prevent the spread and control of outbreaks. The biosecurity measures advised to the different stakeholders are strict quarantine of sick and exposed animals in cases; restriction of animal movements; quarantine of newly purchased animals for at least two to three weeks; decontamination of the premises with common disinfectants; proper disposal of carcasses and contact fomites on-site, restriction on the importation of sheep and goats from affected areas; infected and suspected flocks must be placed under quarantine; personnel should ensure that shoes, clothes, vehicles, and equipment are disinfected. Further, the GoI sponsored PPR-CP has been implemented during FY 2010–2011 to control and eradicate PPR in Southern peninsular India. The strategic vaccination in the program, include mass vaccination (covering the vaccination > 80% of the target population) targeting the defined populations in pulse vaccination mode (to eradicate an epidemic by repeatedly vaccinating a risk population, over a defined age range, in a defined time to quickly stop the spread and contain the outbreak until the spread of the pathogen has been ceased) covering the entire small ruminants population above the age of 4 months old in the designated period followed by annual vaccination covering the 30–40% naïve young population (appearing continuously due to high reproductive rates, and fecundity of small ruminants), to avoid window of susceptibility in kids/lambs to virus infection for 2 years and again mass vaccination to cover the leftover animals in the earlier vaccination programs in each of the states. During 2011 in the first phase, administrative divisions (Karnataka, Andhra Pradesh, Telangana, Tamil Nadu, Kerala, Maharashtra, Goa and Lakshadweep, Daman and Diu, Dadra and Nagar Haveli, Puducherry, and Andaman and Nicobar Island) in Southern peninsular India was covered and the remaining states and UTs were included in the second phase of PPR-CP from 2014 to 2015 [14]. However, this strategic vaccination in the PPR-CP was carried out only in some states of India since 2011. The state-wise details of the number of vaccinations carried out in small ruminants’ population as per the 20th Livestock Census-2019 (except for the undivided AP, where the population was taken as per the 19th Livestock Census-2012) in different zones in India were depicted in Figure 10 along with the FY reported outbreaks in each state. Recently, during 2019, the PPR outbreaks were reported in 16 states and confined to a few districts in the mass vaccinated program implemented states and more districts in the vaccination program not implemented or focused vaccination practiced states. Further, the highest PPR outbreaks were reported from Jharkhand, followed by Maharashtra, Uttar Pradesh, West Bengal, Haryana, Rajasthan, Karnataka, Kerala states, etc. with the highest outbreaks during November to March with a peak during January (Winter season).

### 3.6. Case Studies of the States

**Figure 11:**
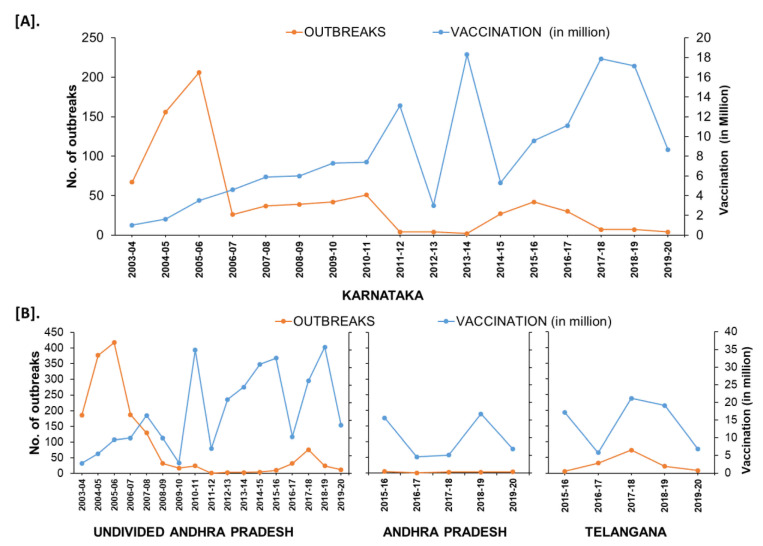
The trend of reduction of outbreaks in the states of Karnataka (**A**) Andhra Pradesh (**B**) from the financial year 2003–2004 to 2019–2020; outbreak reports versus the doses of vaccines used (in million) in the strategic immunizations during the preceding years are depicted as the vaccine produces immunity for 3–6 years.

**Figure 12:**
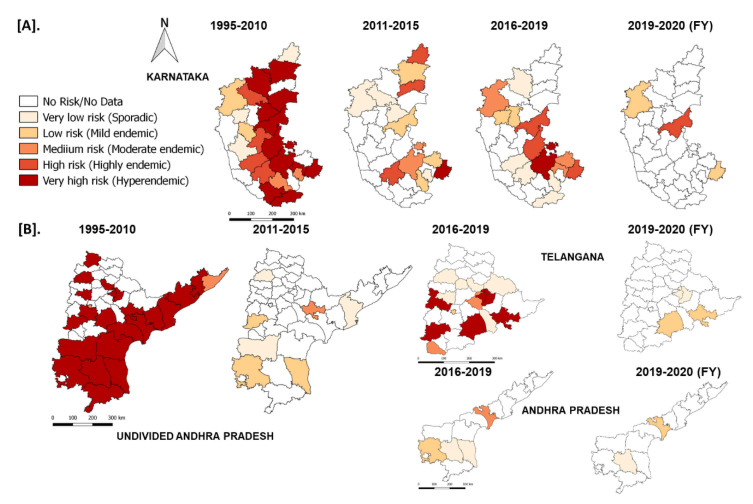
The reported outbreaks in different categories of endemic districts of Karnataka (**A**) Andhra Pradesh (**B**) with risk areas are depicted in six scales at different periods of analysis using QGIS-2.18 in the maps of the respective states. The endemicity categorization is based on the scale of the cumulative outbreaks, that occurred in the districts per year in the given period of analysis and classified into different risk level (if the outbreaks numbers in the district as 0–no risk, 1- very low, 2- low, 3- medium, 4- high and >4 very high-risk districts).

In the South Zone, in Karnataka state, after mass vaccination since 2004, the number of outbreaks declined and reached as low as four outbreaks during the financial year (FY) 2011–2012 & 2012–2013 from 156–206 outbreaks during FY 2004–2006. Further, after adopting the PPR-CP in 2011, the state strategically continued its mass vaccination in the program and covering the entire population (80–90%) within 24 days in pulse vaccination mode. Subsequently, one-third of the naïve population was taken as the target for subsequent vaccination after 6 months, and thereafter every vaccination six-month interval along with leftover animals in previous vaccination. Due to strategic vaccination, the diagnosed PPR cases and deaths reduced, with only four outbreaks during FY 2011–2012 from the reported 206 outbreaks during FY 2005–2006 (Figure 11). As per recent reports from 2017–2020, in Karnataka 4–7 outbreaks were reported in the FY and confined to the three (Belagavi, Bellary, and Kolar) districts (Figure 12). Similarly, in undivided AP (Telangana curved from AP during 2014) state during the year 1999, approximately 552 outbreaks were reported and become persistent with continuously reported outbreaks of ~ 157 to 420 from 2002–2006 every year with a peak of 418 during FY 2005–2006. The undivided AP followed focused vaccination since 2002, and the state implemented a strategic annual vaccination program during FY 2007–2008, with two-cycles of intermittent mass vaccination and selective vaccination to cover the new-born young stock above five months of age and unvaccinated animals to contain the outbreaks and reduced the epidemic level to 95% until 2010. After adopting the PPR-CP, mass vaccination of small ruminants was carried out in pulse vaccination mode followed by bi-annual vaccination (based on the lambing/kidding pattern) to cover naïve young population from 2012 to 2014, which resulted in three outbreaks during FY 2012–2013 & 2013–2014 and reduced the burden by about 99% (one reported outbreak in the FY 2011–2012) against about 377- 418 outbreaks in the FY 2004–2005 & 2005–2006 (Figure 11). In FY 2019–2020, nine outbreaks in three districts (Khammam, Nalgonda, and Warangal-urban) in Telangana state and three outbreaks in Krishna and YSR (Kadapa) districts in Andhra Pradesh were reported (Figure 11). Further, the different categories of endemic districts in Karnataka and undivided AP are depicted in six scale classification at different periods of analysis (Figure 12).

## 4. Discussion

### 4.1. Epidemiological Analysis

In the NADRES database, PPR outbreaks reports are available since 1995 from twenty-two states in the different geographical regions of India and PPR stands first and is the highest among the top 10 reported diseases of small ruminants. However, earlier to 1990, three outbreaks were also reported, of which first one in Tamil Nadu state during 1987 [9] and the other two outbreaks were reported in AP state [26]. Before 1987, RP was believed to be the cause of infection in sheep and goats [27] and whether some of these infections were due to PPR is not clear. The highest reported outbreaks in goats than in sheep with the highest proportion of outbreaks in the south zone states followed by the East, North, West zones states observed in the study was concurrent with various studies by different investigators, from different parts of the world, showing various percentages of mortality and morbidity with the involvement of the different strains of PPRV in both sheep and goats. The varying levels of morbidity and mortality have also been reported in different states of India from 1994 to-date [12,13,14,15,28,29]. Further, the mortality in susceptible flocks varies from 10 to 100% and morbidity from 50% to 100% and sometimes outbreaks can affect an entire flock with 70–90% mortality [30]. However, these proportions may differ in endemic areas where some older animals may have survived an earlier infection. Furthermore, the cumulative PPR report (Figure 5) showed a gradual increase in outbreaks and cases since 1995 and the highest numbers were reported between 2000 and 2007, which probably due to the availability of diagnostic assays since 2002 [11,12,17,31]. Further, the number of reported outbreaks and cases showed a declining trend from 2005, which might be due to the implementation of focussed vaccination in some states since 2002 [11], and the strategic vaccination under PPR-CP in a few states from 2011, besides implementation of the biosecurity measures to prevent the spread and control of PPR outbreaks. However, during 2018–2019, the marginal increase in the number of outbreaks only in some defined geographical areas in Maharashtra, Haryana, Jharkhand, and West Bengal when compared to 2017 (Figure 6), might be due to the migration/movements of the non-vaccinated animals for grazing/trade as well as non-implementation of the vaccination as per the direction of the strategic vaccination under PPR-CP [18,19,20]. Further, the multivariable regression analysis of outbreaks revealed disease occurrence in the East zone was positively associated and significant, as in this zone more outbreaks were being reported than other zones, as extensive vaccination is not being practiced. The decline in outbreaks might be due to the extensive adoption of strategic vaccination in the sheep and goats under the national PPR-CP since 2011 by the major Indian states. In recent years, especially from 2018 onwards the occurrence of PPR in Jharkhand, Maharashtra, West Bengal, Haryana states, had increased, which might be due to the non-implementation of the vaccination or movement of non-vaccinated animals through migrant shepherd in these states. Karnataka and AP have shown a decline in the number of outbreaks during 2011-15 and 2016-19 with sporadic outbreaks in 2018 and 2019; whereas Chhattisgarh state reported no outbreaks since 2013–2014, even though PPR is still reported in some areas of the country [14]. However, West Bengal and Jharkhand states have reported the highest outbreaks during the 2011–2015 and 2016–2019 periods, respectively. 

Seasonal variations (Figure 7) in PPR outbreaks have been recorded in different states in different zones of India. Generally, animal husbandry practices, agro-climatic conditions, and geographical locations affect the seasonal distribution of the disease [12,14,15]. The disease occurs in all seasons but is encountered most frequently during the lean period either in the wet season/ rainy season/ summer or during the cold dry season (December to February) [12] as small ruminants in India are reared on free-range pastureland, shrubs, and forest. The observed significance with Winter season in sheep and goats and Spring and Winter season in sheep was positively associated with the outbreaks, which is probably due to the various causal factors associated with the occurrence of the disease during January to March. In recent years, with the decline in available pastureland and forest area, these animals will often travel long distances during the dry season in search of fodder and water. Temporally, most outbreaks were observed during the Winter and early fall (November to March) than other seasons. On the commencement of monsoon, an increase in the availability of local fodder restricts migration of animals which results in a substantial decrease in the frequency of outbreaks in the sub-Himalayan region as well as in dryland areas (Rajasthan and Gujarat states) as reported earlier [12]. Similar observations were also made during a five-year study of PPR in the tropical humid zone of southern Nigeria [32]. The increased animal trade/the movement of animals during December, lambing seasons, seasonal environmental conditions/summer or wet season or lean period (animals are usually under stress due to long-distance traveling and nutritional deficiency) might be the epidemiological or risk factors associated with the occurrence of the disease [12,15,33,34,35]. During their migration, infected animals may transmit the virus to susceptible local sheep and goats [12,35]. All these speculated observations need further detailed research for a better understanding of the associated epidemiological parameters. Furthermore, climatic factors favorable for the survival and spread of the virus may also contribute to the seasonal distribution of PPR outbreaks in different geographical regions. With the start of the rainy season (between June/July and August/September), the migratory activity of animals is reduced due to the increased availability of local fodder. The nutritional and health status of the animals also improves, resulting in increased resistance to infection. Most of the investigators have invariably linked the PPR outbreak with the introduction of new animals to the flocks [12,36,37,38,39] or major festival time involving greater movements of animals. Consequently, large numbers of animals become infected and then help to maintain the circulation of the virus throughout the year by the frequent animal to animal transmission [12]. Therefore, three weeks quarantine of new animals in the farm might prevent the occurrence of PPR. In conclusion, the most appropriate time to vaccinate against PPR is August/September and March/April in a year and well before the migration of the animals.

In the present study, it was observed that the highest 47% outbreaks (with 54% cases) were reported in goats followed by 43% in sheep (with 23% cases), with an increased number of outbreaks in goats than in sheep in the different zones of the country, except in the south zone where the number of outbreaks was higher in sheep. This could be due to PPR affects goats more than sheep and the population of goats to sheep is almost 2:1 in India as per the 20th livestock census 2019 [8] and in South-zone, the population of sheep is higher than goats in Andhra Pradesh, Telangana, and Karnataka states. Outbreaks are relatively more common in goats than sheep in northern India [12,40,41], as the goat population is more in northern India [12]. The higher rate of slaughtering of male goats at an early age and high reproductive rates, and fecundity of goats results in the appearance of the naïve population continuously [14,28] and therefore naïve population becomes susceptible to the infection; presumably, another reason for greater susceptibility of the goats [12,15]. Further, it was observed that deaths were reported more in goats (60%) than in sheep (23.98%) with the highest proportion in the East zone (46%) followed by the South zone (26%), which is corroborated with most of the earlier reports stating that though the PPRV infects both sheep and goats, the severity of the clinical symptoms was more predominant in goats than sheep [12] and goats were severely infected than sheep [12,13,15]. Similarly, Soundararajan et al. [36] reported a higher mortality rate among infected goats than sheep in a large organized farm. The vaccination program implemented in many states had reduced the deaths due to PPR significantly. In AP/Telangana state the reported CFR declined from 61.0% to 32.3% during 2011–2015 to 2016–2019 (Figure S1). In general, infected sheep and goats on subsequent recovery from the disease are protected from re-infection for their life. Recovery rates from PPRV infection are considerably lower for goats than for sheep, resulting in a low proportion of the goat population being protected from re-infection [16,28]. Further, this could be due to differences in the virulence of field strains for both species or sheep might have an innate resistance to clinical development of the disease [37]. Furthermore, different host genetics and non-genetic factors may play a significant role in variation to disease susceptibility [42]. In the laboratory studies, some of the PPR viruses isolated from goats from north India have been shown to cause severe infection in goats as compared to sheep [12,41]. Curiously, some of the PPR outbreaks attended by our investigation team in Karnataka state found more pronounced clinical signs and severe disease in sheep than goats in flocks where sheep and goats reared together. However, a report also indicates that outbreaks are more common in sheep than goats in southern India [43]. Thus, a gradual adaptation of the virus in particular species, species selection of strains, and further amplification through numerous natural passages in the population of the regions may lead to adaptation to a particular species resulting in more severe disease in adapted species in the particular region. Moreover, this scenario is likely to change drastically once intensive vaccinations are carried in the small ruminants’ population [20]. Therefore, the studies at the molecular aspects of viral determinants would unravel the species susceptibility levels. However, it cannot be undermined that, the recovery rate in goat is comparatively less than that in sheep after infection. Nevertheless, further experimental infection studies are required to study virus characteristics and species susceptibility at the molecular level to understand the underlying mechanism to map both viral and genetic makers of differential disease severity in sheep and goats. 

On spatial analysis of outbreaks data (Figure 9), PPR endemic risk areas showed a wide variation in the different states/zones of India at different periods. Based on the occurrence of the cumulative outbreaks in the endemic districts, the top three districts in different categories (hyperendemic, highly endemic, low endemic, and sporadic) in different states of India were obtained and are summarised (Table S2). The variation in disease endemicity might be due to differences in animal husbandry practices and the agro-climatic conditions affecting the pattern of the natural vegetation which indirectly influences the socio-economic factors, the migration patterns of small ruminants, flock size, and the population density of the animals in the different states. Although India is endemic to PPR, north-eastern states either free from disease or have very few reports [12,21,44]. North-East states have a relatively small sheep and goats population and intermixing of these animals with the small ruminants population from the rest of the country is usually limited because of very narrow connecting passage [12,21,44]. Further, the hilly terrain characterizing this region may restrict the movement of animals and disease transmission [45]. 

### 4.2. PPR Control Program 

PPR control depends mainly on accurate diagnosis, surveillance/monitoring, and effective implementation of the vaccination program. The choices of control strategies in developing or under-developed countries are limited. Rigorous stamping out policy involving quarantine and slaughter can control the spread of the disease and aid in eradication, but difficult to follow in developing countries like India due to various socio-economic and sentimental reasons. However, in the final stage of eradication, elimination of the virus is possible through slaughter or restriction in the movement of animals. Generally, social acceptance, public and regulatory support is essential for the success of the disease control and eradication program. Hence, vaccination is a recommended tool to support control and eradication efforts [11,28]. 

#### 4.2.1. Vaccination 

In India, live attenuated PPR vaccine (Sungri-96 strain–lineage IV virus) developed by Indian Veterinary Research Institute (IVRI), had undergone extensive field trials [11,46]. This experimental PPR vaccine after field testing has been administered/practiced as focused vaccination during outbreaks to control and prevent the disease spread in 15 states of India since 2002 [11]. Seroconversion and protection have been observed in vaccinates [11] with a field vaccine dose of 10^3^ TCID_50,_ and protective immunity is ensured for >6 years without a booster [47], and this vaccine is well suited for mass immunization program [14]. The vaccine production and quality control technology has been transferred to different national and multinational companies in India and Veterinary Biological Production Units/Institute of Animal Health and Veterinary Biologicals of different states of India. PPRV is a single serotype virus, and hence, any vaccine lineage virus can protect against all other field viruses/ field isolates/strains of PPRV lineages [48] and provide complete clinical protection against challenges with all four lineages of PPRV [49]. Therefore, this vaccine can be used for the control and eradication of the disease not only from India but also from other countries following the PPR-GEP. The availability of an effective vaccine, accurate mass screening diagnostic assays, an experienced/improved infrastructure, expertise, success with the eradication of RP has provided confidence and prompted India to propose a national PPR-CP on the lines of a national program on RP eradication without much additional budgetary encumbrance. All the aforesaid available elements [12,28] guided the policymakers to initiate a nation-wide PPR-CP under the direction of the DAHD, GoI to the State Animal Husbandry Departments. A technically feasible, economically viable, and practically attainable proposition of PPR-CP has been implemented during 2010–2011 to control and eradicate PPR from India [13] by PPR mass vaccination [14]. During the first phase, vaccination was covered in Southern peninsular India and the remaining states and UTs were included in the second phase of PPR-CP from 2014 to 2015 [14]. 

A strategic vaccination of the population to attain 80% herd immunity would be needed to account for the population dynamics of small ruminants, differences in animal husbandry practices, and the agro-climatic conditions affecting the pattern of disease [28]. Initially, ‘intensive mass vaccination’ of the entire population within a specified time, subsequently ‘vaccinations on younger animals’ is necessary to avoid window of susceptibility in kids/ lambs to PPRV infection [14]. The vaccinated animals are protected from re-infection for the remainder of their lives. Hence, in this direction, the strategy vaccination of the PPR-CP involves intensive mass vaccination of all susceptible sheep and goats above 5–6 months age group of animals in pulse vaccination mode, and two successive annual vaccination of their subsequent generations (30–40% naïve young population every year) [50] to reach 70–80% immunity level and again mass vaccination of the entire sheep and goats population in each of the states. This strategy is to cover the naïve population appearing in the flock continuously due to high reproductive rates, fecundity, and slaughtering of male goats at an early age [14,28]. 

On analysis, among the zones, the effect of vaccination was more effective and pronounced in the South zone resulted in a drastic reduction in the reported outbreaks and cases. Karnataka state reported the disease for the first time in 1992 [34] it progressed later across varied agro-climatic conditions with varying intensities and reached a peak during 2004–2006 [35]. Since 2004, the state followed mass vaccination, and in consonance with PPR-CP during 2011, which resulted in a decline in the number of outbreaks. Further, the undivided AP followed focused vaccination since 2002 to contain the outbreaks and reduced the epidemic level by 95% [29], and the state implemented a mass vaccination during 2007–2008 and followed annual programs until 2010 [51] and in consonance with PPR-CP from 2012 to 2014, which reduced the burden by about 99% with the flock immunity of 81 to 95.6% [14,29]. This is due to systemic vaccination programs followed in these states during the first phase of PPR-CP [20]. In the Central zone, Chhattisgarh state-initiated the PPR annual vaccination program (as mass vaccination campaign) since 2010 on the lines of ‘pulse polio program’ in the designated period (11–12 days) with a mass media campaign to reach out to livestock farmers [14] resulting in no report of PPR since 2013–2014 [52]. Besides farm-reared animals, goat markets, nomadic and selling units, check posts, etc., were also vaccinated to maximize the vaccination coverage. Through a strategic annual vaccination program in pulse vaccination mode, PPR has been kept under control in the state [50] and it may eventually assist in its eradication. The strategic vaccination has been systematically followed (Karnataka, Andhra Pradesh/Telangana, and Chhattisgarh) since the beginning of PPR-CP and these states control the disease and reduced the epidemics successfully, and it may eventually lead to eradication [14]. Further, the state has initiated the policy measures to implement focused ring vaccination in the area of the outbreak along with the biosecurity measures to contained outbreaks. In the recent past, since 2015 in the second phase of PPR-CP, most of the states and UTs in India followed strategic vaccination, thereby the reported overall outbreaks and disease threat have been reduced significantly in India. Due to PPR-CP, the disease has been brought under control in Indian states and the disease threat declined progressively and substantially in areas under continuous vaccination [14] and benefits outweigh the cost of a vaccination program [50]. The PPR outbreaks trend at the national level showed about 75–80% decline [29], however, there was no further definite declining trend during the years (2009–2013) [14]. However, in some states, where focused/targeted vaccination is adopted, disease outbreaks are being reported sporadically. Generally, the success of the vaccination and control of disease depends on various factors, like animal husbandry practices, population density, lambing and kidding seasons, movement / migratory population of animals, etc., including the observed vaccination-related field problems, like vaccine-chain mechanism, identification of unvaccinated animals, farmers insistence on repeat vaccination and cold chain maintenance for storage of vaccines, timely supply of the vaccine, and wastage of vaccines in the field due to high package vaccine doses supplied, etc., [14,51]. All these factors and constraints need to the addressed for the success of the program, along with the adoption of biosecurity measures. 

#### 4.2.2. Seromonitoring and Surveillance

Studying the prevalence of PPRV antibodies in susceptible hosts from different geographical areas with varying agro-climatic conditions may help to devise appropriate disease control strategies [16]. Since 2002, focused vaccination and/or strategic vaccination under PPR-CP were carried out in some states but neither systematic sero-monitoring was initiated during the last decade to assess the efficacy of the vaccination program nor any sero-surveillance plan was undertaken during these periods [29]. A national-wide serosurvey in sheep and goats during 2017–2018 by PPR competitive ELISA [17] showed wide variations of PPR seroprevalence/immune population in the different states in India [18,19,20,21]. The immune populations were greater in regular vaccination practiced states (Andhra Pradesh, Telangana, Karnataka, Chhattisgarh, Maharashtra, Gujarat, Punjab, Haryana) compared with focused or non-vaccinated states (Kerala, Tamil Nadu, Bihar, Assam, Goa, Himachal Pradesh, Jammu and Kashmir, Madhya Pradesh, Manipur, Meghalaya, Mizoram, Nagaland, Odisha, Tripura, Uttara Pradesh, Uttarakhand, West Bengal), where the disease is endemic and outbreaks are being regularly reported. By employing competitive ELISA, it is not possible to distinguish the vaccinated and infected animals, as the DIVA vaccine was not being used in the PPR-CP. However, the earlier surveys in the non-outbreaks reported state (Chhattisgarh) indicated above 50% prevalence of PPRV antibodies indicates vaccination is being implemented [53]. Further, the baseline seroprevalence in sheep and goats before the implementation of the mass vaccination varied from 32.4 to 46.11% [12,16]. Further, in Sikkim state, Andaman and Nicobar Islands, and other isolated niches, vaccination is not required to be implemented, because the PPRV has not yet become established [45,54] despite the endemic nature of the disease in the rest of India due to its unique geographical location. However, recently, Sikkim state had reported 15 diagnosed cases with one death out of 100 susceptible risk population from one sporadic outbreak during 2018-19 and the state had initiated the vaccination covering the small ruminant (~39,900) population. Therefore, this implies the need for legal frameworks, strict quarantine measures, and restrictions in the small ruminants’ movements and trade along with intensive active surveillance program to monitor sporadic outbreaks and to achieve disease freedom and move from eradication stage 1 to stage 4, as per the PPR GCES in the isolated geographical regions/niche. 

Further, the vaccination strategies adopted in the region or geographical location will alter PPR epidemiology particularly distribution and pattern of disease [12,15,55] ie., decreased numbers of outbreaks in general and disease severity pattern in particular, as changing pattern in term of the severity of gross lesions and clinical signs recently observed in the region, where vaccination was regularly carried out [20,21,56,57]. Further, on cumulative analysis of the cases and deaths in undivided AP revealed the highest deaths of sheep and goats (n = 17,175) during 1995–2010, had declined during 2011-15 (n = 180) and 2016-19 (n = 269), which implies the changing pattern of the disease with less severity due to ongoing vaccination in the state since inception as the observed case fatality rate was declined from 61 to 32% during 2011–2015 to 2016–2019. This might be due to the effectiveness, timely vaccination of sheep and goats, in a few states of India [2]. In the later stage of the disease control plan by mass vaccination strategies, the disease conditions may not always produce all the classical clinical signs of PPR in the animals during the sporadic outbreaks. Hence, during the eradication stage, syndromic surveillance is needed to recognize and identify the mild form of the disease in all possible cases [57]. This will help in deprived of the hindrance of surveillance in the last phase of eradication in the PPR-GEP and avoid delay in the declaration of the provisionally free status of PPR. Further, there is a need for a disease registry, both at the state and at national levels, to ensure effective disease reporting and coordination of outbreak occurrence and surveillance during the eradication phase. This would help in providing a rapid appraisal of the movement of animals, and tracking and locating prospective animal target foci, early detection or identification of the pathogen, and prompt initiation of control and biosecurity measures. Further, creating awareness on biosecurity among the rural communities, animal movement management, and professional commitment on the part of veterinarians and ancillary personnel involved in the immunization program is crucial at the eradication stage. 

#### 4.2.3. National Strategic Plan for PPR Eradication 

Zoning the PPR risk regions and initiating the strategic vaccination program at a specified period with high coverage of mass vaccination of all the targeted risk population is required to eliminate PPR in the identified epi-zone along with monitoring and surveillance to control and eradicate PPR. This necessitates intensive mass vaccination implementation [14,29], by the state animal husbandry and veterinary service, as animal husbandry is a state subject. In this direction, the Department of Animal Husbandry and Dairying, Ministry of Fisheries, Animal Husbandry and Dairying, GoI, prepared the National strategic plan for PPR Eradication 2025 in the lines of the National Rinderpest Eradication Program to eradicate PPR by 2030 in consonance with the PPR GCES. The salient features of the plan include strategic vaccination with complete coverage of sheep and goats’ populations till 2022, attaining targeted herd immunity and stoppage of virus circulation through clinical surveillance by 2023/24, and freedom from PPRV infection by 2025. The strategic vaccination in the program, include mass vaccination will be targeting the defined populations in pulse vaccination mode in the designated period followed by annual vaccination covering the 30–40% naïve young population for 2 years to reach 70–80% immunity level, and again mass vaccination of the population to cover the leftover animals in the earlier vaccination programs in each of the states. Epi-zone or risk zone /area needs to be identified in the state and vaccination to be initiated first in the high-risk area followed by the row risk area in the various districts of the state concerning the risk/target population. State-level departmental units should plan for pre-and post-vaccination sero-monitoring during the program period, including training of field veterinarians and vaccinators and supporting staff. Overall monitoring including the real-time data flow from the field level, procurement of vaccine, storage, shipments, cold chain maintenance, and other vaccine-chain mechanism and logistics till the delivery of the vaccine to the animals through different departmental units of the state will provide effective implementation of the program in India. 

The present study had some limitations; firstly, only the reported outbreaks/cases were considered for epidemiological analysis and PPR outbreaks were likely under-reported due to lack of machinery to collect the real-time field situation owning to inadequate animal disease surveillance and reporting system. Secondly, most of the reported earlier outbreaks before the development of the ELISA diagnostics in India, i.e., before 2005, were based on classical signs of PPR by professional field veterinarians and not based on laboratory confirmation of PPR, as the OIE lists many other diseases as a differential diagnosis to PPR, like Contagious caprine pleuropneumonia, Bluetongue, Pasteurellosis, Contagious ecthyma, FMD, etc. [58]. Lastly, no data is available on the status of breed, sex, age of the animals involved in the outbreaks for further analysis.

## 5. Conclusions 

The present study provides insight on the temporal patterns and spatial distribution of PPR in India as well as disease burden, host specificity, status of the control program, and time of outbreaks/cases to support policymakers to take appropriate decisions. It also identifies when and where intensive surveillance and vaccination effective control strategies could be implemented more efficiently for the control and eradication of PPR in different regions/zones of India. At present, the disease has been brought under control by effective and safe live attenuated PPR vaccine, but only with effective implementation of a strategic vaccination, complete control and eradication of the disease from India is possible. Sharing experiences on the vaccination strategies adopted by some states in India may motivate other states or countries with similar geographical size and socio-economic and/or animal husbandry practices for similar initiatives (strategies conducive and highly suitable based on their available resources and facilities, etc.) leading to control of PPR. Further, the central, state, and the district-level technical working group on various elements of PPR control and eradication viz., diagnostics, surveillance, disease prevention, and control, legal framework, and involvement of different stakeholders need to be established in the national strategic plan for PPR eradication in the direction of PPR-GEP for overall monitoring and effective implementation of the program and successful eradication of PPR from India. 

## Data Availability

All datasets supporting our findings are available at the Spatial Epidemiology Laboratory of the ICAR-NIVEDI, Bengaluru on reasonable request from the corresponding author or co-authors.

## Supplementary Materials

The following are available online at https://www.mdpi.com/1999-4915/13/3/480/s1.
