# Supplementary material for: Temporal and Spatial Epidemiological Analysis of Peste Des Petits Ruminants Outbreaks from the Past 25 Years in Sheep and Goats and Its Control in India
Source: Viruses. 2021 Mar 15;13(3):480. doi: 10.3390/v13030480 (PMC8001942; doi:10.3390/v13030480)

Supplementary Information

Temporal and spatial epidemiological analysis of peste des petits ruminants outbreaks from the past 25 years in sheep and goats and its control in India

Table S1. Estimated multivariable linear regression coefficients of important parameters associated with PPR outbreaks in sheep and goats

[A]. Sheep

Parameters	β	SE	t-value	p-value		95 % Confident Interval	
						Lower limit	Upper limit
(Intercept)	-4.844	4.861	-0.997	0.319		-14.371	4.683
East zone	-2.352	2.764	-0.851	0.395		-7.768	3.065
North zone	-0.905	2.764	-0.327	0.743		-6.322	4.512
North-East zone	-5.792	5.263	-1.101	0.271		-16.108	4.524
South zone	18.255	2.764	6.605	0.000	***	12.838	23.672
West zone	-2.038	2.764	-0.738	0.461		-7.455	3.378
Monsoon season	-0.784	2.854	-0.275	0.784		-6.378	4.810
Pre-winter season	0.176	2.854	0.062	0.951		-5.418	5.770
Spring season	6.912	2.854	2.422	0.016	**	1.318	12.506
Summer season	-0.544	2.854	-0.191	0.849		-6.138	5.050
Winter season	15.910	3.061	5.198	0.000	***	9.911	21.909
1996	-0.100	5.826	-0.017	0.986		-11.520	11.320
1997	-0.100	5.826	-0.017	0.986		-11.520	11.320
1998	2.100	5.826	0.360	0.719		-9.320	13.520
1999	5.367	5.826	0.921	0.357		-6.053	16.786
2000	17.100	5.826	2.935	0.003	***	5.680	28.520
2001	1.800	5.826	0.309	0.757		-9.620	13.220
2002	7.667	5.826	1.316	0.189		-3.753	19.086
2003	9.633	5.826	1.653	0.099		-1.786	21.053
2004	12.467	5.826	2.140	0.033	**	1.047	23.886
2005	18.700	5.826	3.210	0.001	***	7.280	30.120
2006	8.867	5.826	1.522	0.128		-2.553	20.286
2007	4.933	5.826	0.847	0.397		-6.486	16.353
2008	2.567	5.826	0.441	0.660		-8.853	13.986
2009	0.233	5.826	0.040	0.968		-11.186	11.653
2010	1.033	5.826	0.177	0.859		-10.386	12.453
2011	-0.367	5.826	-0.063	0.950		-11.786	11.053
2012	-0.433	5.826	-0.074	0.941		-11.853	10.986
2013	-0.633	5.826	-0.109	0.913		-12.053	10.786
2014	-0.233	5.826	-0.040	0.968		-11.653	11.186
2015	0.067	5.826	0.011	0.991		-11.353	11.486
2016	0.900	5.826	0.154	0.877		-10.520	12.320
2017	0.200	5.826	0.034	0.973		-11.220	11.620
2018	1.967	5.826	0.338	0.736		-9.453	13.386
2019	0.367	5.826	0.063	0.950		-11.053	11.786

Note: β-coefficient; SE-Standard Error; Significant *** at 1 % ($p < 0.01$); ** at 5 % ($p < 0.05$) level.

Multiple R-squared: 0.207, F-statistic: 5.488, p-value: $< 2.2e-16$

[B].Goats.

Parameters	B	SE	t-value	p-value		95 % Confidence interval	
						Lower limit	Upper limit
(Intercept)	-1.289	2.128	-0.606	0.545		-5.459	2.882
East zone	17.027	1.246	13.666	< 2e-16	***	14.585	19.469
North zone	0.373	1.246	0.300	0.765		-2.069	2.815
North-East zone	-0.520	1.246	-0.417	0.677		-2.962	1.922
South zone	3.533	1.246	2.836	0.005	***	1.091	5.975
West zone	-0.027	1.246	-0.021	0.983		-2.469	2.415
Monsoon season	-0.920	1.246	-0.738	0.460		-3.362	1.522
Prewinter season	-0.053	1.246	-0.043	0.966		-2.495	2.389
Spring season	-0.620	1.246	-0.498	0.619		-3.062	1.822
Summer season	-1.513	1.246	-1.215	0.225		-3.955	0.929
Winter season	0.453	1.246	0.364	0.716		-1.989	2.895
1996	-1.250	2.543	-0.492	0.623		-6.235	3.735
1997	-1.250	2.543	-0.492	0.623		-6.235	3.735
1998	2.333	2.543	0.917	0.359		-2.651	7.318
1999	-1.083	2.543	-0.426	0.670		-6.068	3.901
2000	0.028	2.543	0.011	0.991		-4.957	5.012
2001	2.750	2.543	1.081	0.280		-2.235	7.735
2002	-0.028	2.543	-0.011	0.991		-5.012	4.957
2003	4.111	2.543	1.617	0.106		-0.874	9.096
2004	1.528	2.543	0.601	0.548		-3.457	6.512
2005	9.472	2.543	3.725	0.000	***	4.488	14.457
2006	5.000	2.543	1.966	0.050	**	0.015	9.985
2007	5.056	2.543	1.988	0.047	**	0.071	10.040
2008	1.500	2.543	0.590	0.555		-3.485	6.485
2009	1.750	2.543	0.688	0.492		-3.235	6.735
2010	3.750	2.543	1.475	0.141		-1.235	8.735
2011	3.750	2.543	1.475	0.141		-1.235	8.735
2012	11.333	2.543	4.456	0.000	***	6.349	16.318
2013	3.722	2.543	1.464	0.144		-1.262	8.707
2014	2.083	2.543	0.819	0.413		-2.901	7.068
2015	3.444	2.543	1.354	0.176		-1.540	8.429
2016	2.944	2.543	1.158	0.247		-2.040	7.929
2017	-0.833	2.543	-0.328	0.743		-5.818	4.151
2018	2.944	2.543	1.158	0.247		-2.040	7.929
2019	2.056	2.543	0.808	0.419		-2.929	7.040

Note: β-coefficient; SE-Standard Error; Significant *** at 1 % ($p < 0.01$); ** at 5 % ($p < 0.05$) level.

Multiple R-squared: 0.3011, F-statistic: 10.96, p-value: < 2.2e-16

[C]. Sheep and Goats combined

Parameters	B	SE	t-value	p-value		95% Confident interval	
						Lower limit	Upper limit
(Intercept)	-7.474	4.935	-1.515	0.130		-17.146	2.197
East zone	18.373	2.889	6.359	0.000	***	12.710	24.036
North zone	3.067	2.889	1.061	0.289		-2.596	8.730
North-East zone	-0.573	2.889	-0.198	0.843		-6.236	5.090
South zone	25.927	2.889	8.973	< 2e-16	***	20.264	31.590
West zone	2.060	2.889	0.713	0.476		-3.603	7.723
Monsoon season	-1.647	2.889	-0.570	0.569		-7.310	4.016
Pre-winter season	0.553	2.889	0.192	0.848		-5.110	6.216
Spring season	5.613	2.889	1.943	0.052		-0.050	11.276
Summer season	-1.880	2.889	-0.651	0.515		-7.543	3.783
Winter season	12.853	2.889	4.449	0.000	***	7.190	18.516
1996	-1.306	5.898	-0.221	0.825		-12.865	10.254
1997	-0.722	5.898	-0.122	0.903		-12.282	10.837
1998	8.806	5.898	1.493	0.136		-2.754	20.365
1999	5.389	5.898	0.914	0.361		-6.171	16.949
2000	15.167	5.898	2.572	0.010	**	3.607	26.726
2001	4.028	5.898	0.683	0.495		-7.532	15.587
2002	6.472	5.898	1.097	0.273		-5.087	18.032
2003	14.000	5.898	2.374	0.018	**	2.440	25.560
2004	11.722	5.898	1.988	0.047	**	0.163	23.282
2005	24.250	5.898	4.112	0.000	***	12.690	35.810
2006	12.028	5.898	2.039	0.042	**	0.468	23.587
2007	8.722	5.898	1.479	0.140		-2.837	20.282
2008	3.167	5.898	0.537	0.592		-8.393	14.726
2009	1.667	5.898	0.283	0.778		-9.893	13.226
2010	3.917	5.898	0.664	0.507		-7.643	15.476
2011	2.778	5.898	0.471	0.638		-8.782	14.337
2012	10.139	5.898	1.719	0.086	.	-1.421	21.699
2013	2.639	5.898	0.447	0.655		-8.921	14.199
2014	1.278	5.898	0.217	0.829		-10.282	12.837
2015	3.500	5.898	0.593	0.553		-8.060	15.060
2016	3.167	5.898	0.537	0.592		-8.393	14.726
2017	-1.250	5.898	-0.212	0.832		-12.810	10.310
2018	3.972	5.898	0.674	0.501		-7.587	15.532
2019	2.111	5.898	0.358	0.721		-9.449	13.671

Note: β-coefficient; SE-Standard Error; Significant *** at 1 % ($p < 0.01$); ** at 5 % ($p < 0.05$) level.

Multiple R-squared: 0.217, F-statistic: 7.053, p-value: < 2.2e-16

Table S2. Different categories of major endemic districts in different states of India*

<table>
<tr><th rowspan="2">Names of States</th><th colspan="3">District endemicity (1995-2010)</th><th colspan="3">District endemicity (2011-2015)</th><th colspan="3">District endemicity (2016-2019)</th></tr>
<tr><th>Hyperendemic</th><th>Endemic</th><th>Sporadic</th><th>Hyperendemic</th><th>Endemic</th><th>Sporadic</th><th>Hyperendemic</th><th>Endemic</th><th>Sporadic</th></tr>
<tr><td colspan="10">SOUTH ZONE</td></tr>
<tr><td>Andhra Pradesh</td><td rowspan="2">Adilabad, Anantapur, Chittoor, East Godavari, Guntur, Kadapa(YSR), Karimnagar, Khammam, Krishna, Kurnool, Mahabubnagar, Medak, Nalgonda, Nizamabad, Prakasam, Rangareddy, Sri Potti Sriramulu Nellore, Visakhapatnam, Vizianagaram, Warangal, West Godavari</td><td rowspan="2">Hydrabad, Srikakulam</td><td rowspan="2"></td><td rowspan="2"></td><td rowspan="2">Khammam, Mahabubnagar</td><td rowspan="2">Kurnool, Nizamabad</td><td></td><td>Anantapur Krishna</td><td>Sri Potti Sriramulu Nellore</td></tr>
<tr><td>Telangana</td><td>Khammam, Mahabubnagar, Nalgonda, Sangareddy, Warangal</td><td>Hydrabad, Jangaon, Jogulamba, Yadadri</td><td>Jayashankar, Karimnagar, Medak, Nizamabad, Rajanna Sircilla Suryapet</td></tr>
<tr><td>Karnataka</td><td>Bangalore Rural Bellary, Bijapur Chamarajanagar, Chitradurga, Gulbarga, Kolar</td><td>Bagalkot, Bangalore, Belgaum, Chikmagalur, Davanagere, Hassan, Haveri, Mandya</td><td>Bidar, Dharwad, Shimoga</td><td>Kolar</td><td>Bellary, Bidar, Chikkaballapura, Gulbarga, Hassan Ramanagara, Tumkur, Yadgir</td><td>Bagalkot, Bangalore, Belgaum, Koppal,</td><td>Chamarajanagar, Tumkur</td><td>Bangalore Rural, Belgaum, Bellary, Chikkaballapura, Chitradurga, Dharwad, Gadag, Kolar</td><td>Bijapur, Chikmagalur, Hassan, Ramanagara</td></tr>
</table>

	Koppal, Mysore, Raichur, Tumkur								
**Kerala**			Palakkad	Palakkad	Alappuzha, Ernakulam, Idukki, Kollam, Kozhikode, Malappuram, Thiruvananthapuram, Thrissur	Kannur, Pathanamthitta, Wayanad,		Alappuzha, Kasaragod, Kozhikode, Malappuram, Palakkad, Thiruvananthapuram, Thrissur	Ernakulam Kollam
**Puducherry**						Puducherry			
**Tamil Nadu**		Cuddalore, Dindigul, Erode, Salem, Thanjavur, Tirunelveli, Vellore	Dharmapuri, Madurai, Nagapattinam, Namakkal, Pudukkottai, Ramanathapuram, Thiruvallur, Thiruvarur, Tiruvannamalai		Dindigul, Kancheepuram, Salem, Nilgiris, Thoothukkudi, Tiruppur, Vellore, Viluppuram,	Dharmapuri, Kanniyakumari, Karur, Namakkal, Sivaganga, Thanjavur, Tiruchirappalli, Tirunelveli, Tiruvannamalai, Virudhunagar		Cuddalore	Coimbatore, Tiruppur
**NORTH ZONE**									
**Haryana**	Hisar, Sirsa		Bhiwani			Bhiwani, Hisar, Kaithal	Hisar	Bhiwani, Sirsa	Fatehabad, Gurgaon, Hanumangarh, Jhajjar, Kaithal, Sonipat
**Himachal Pradesh**	Chamba, Kangra, Kinnaur, Kullu, Mandi, Shimla	Hamirpur, Lahul & Spiti, Sirmaur, Solan			Solan	Bilaspur, Kinnaur, Shimla		Chamba, Shimla	Hamirpur, Kangra, Kullu, Lahul & Spiti, Mandi, Solan
**Jammu & Kashmir**	Jammu, Udhampur	Doda, Kathua, Leh(Ladakh), Punch, Rajouri	Badgam, Ganderbal, Pulwama			Badgam, Pulwama		Baramula, Kupwara	Pulwama
**Punjab**		Ludhiana	Bathinda, Jalandhar,			Jalandhar, Ludhiana,		Ludhiana	Barnala

			Kapurthala, Sangrur			Patiala			
**Uttar Pradesh**					Bareilly	Bulandshahr	Bareilly, Gonda, Gorakhpur	Etah, Fatehpur, Hardoi, Kanpur Nagar	Bara Banki, Hamirpur, Kushinagar, Meerut, Sambhal, Sitapur, Unnao
**WEST ZONE**									
**Gujarat**	Bhavnagar, Jamnagar, Junagadh, Rajkot	Banas Kantha, Bhavnagar, Jamnagar, Junagadh, Rajkot	Amreli, Kachchh, Kheda		Amreli, Bhavnagar, Rajkot, Valsad	Gandhinagar, Junagadh, Kheda, Navsari, Sabar Kantha		Amreli, Rajkot, Tapi	Ahmadabad, Bhavnagar, Gandhinagar, Junagadh
**Maharashtra**	Nashik	Ahmadnagar, Dhule, Jalgaon, Kolhapur, Nagpur, Osmanabad, Pune, Sangli	Aurangabad, Buldana, Chandrapur, Gondiya, Jalna, Latur, Parbhani, Raigarh, Satara, Sindhudurg, Solapur	Ahmadnagar, Solapur	Aurangabad, Bid, Gadchiroli, Jalgaon, Nagpur, Nashik, Osmanabad, Pune,	Latur, Sangli	Ahmadnagar, Dhule Nashik	Gadchiroli	Jalgaon, Nagpur, Pune, Thane, Wardha
**Rajasthan**	Churu	Ajmer, Kota, Sikar	Alwar, Bhilwara, Bundi, Jaipur, Jhunjhunun, Sawai Madhopur, Udaipur,		Baran, Kota, Nagaur, Sikar, Tonk, Udaipur	Bhilwara, Dausa, Dhaulpur, Jaipur, Jaisalmer, Jodhpur, Pali		Churu, Jaipur, Jaisalmer, Jodhpur, Sawai Madhopur	Baran, Hanumangarh, Jhalawar, Kota, Pali
**EAST ZONE**									
**Bihar**		Bhojpur, Buxar, Lakhisarai, Munger, Patna	Bhagalpur, Jamui			Gaya, Muzaffarpur			Arwal, Aurangabad, Bhojpur, Gaya, Jehanabad, Kaimur (Bhabua), Muzaffarpur, Purba Champaran, Siwan, Supaul

**Jharkhand**	Palamu	Bokaro, Dhanbad, Garhwa, Giridih, Gumla, Hazaribagh, Lohardaga, Purbi, Singhbhum, Ranchi	Chatra, Dumka, Godda, Pakur, Sahibganj	Bokaro, Dhanbad, Pakur, Ranchi	Chatra, Deoghar, Godda, Hazaribagh, Jamtara, Kodarma, Latehar, Lohardaga	Dumka, Gumla, Palamu, Pashchimi, Singhbhum, Purbi, Singhbhum, Saraikela-Kharsawan	Bokaro, Dumka, Khunti, Kodarma, Lohardaga, Sahibganj, Simdega	Deoghar, Dhanbad	Godda, Palamu, Purbi Singhbhum Saraikela-Kharsawan
**Odisha**	Anugul, Kalahandi, Kandhamal, Kendujhar, Koraput, Nuapada, Rayagada	Bargarh, Baudh, Cuttack, Debagarh, Ganjam, Khordha, Malkangiri, Mayurbhanj, Nayagarh, Sambalpur, Sundargarh	Jajapur	Kendrapara	Baleshwar, Dhenkanal, Ganjam, Kendujhar, Khordha, Nabarangapur, Nuapada, Sambalpur	Balangir, Baudh, Gajapati, Jajapur, Kalahandi, Kandhamal, Mayurbhanj, Nayagarh, Rayagada	Ganjam	Jagatsinghapur, Khordha, Mayurbhanj	Baudh, Cuttack, Debagarh, Dhenkanal, Kendrapara, Kendujhar, Malkangiri, Sambalpur
**West Bengal**	Bankura, Barddhaman, Birbhum, Dakshin, Dinajpur, Hugli, Jalpaiguri, Maldah, Nadia, Paschim, Medinipur, Puruliya, South Twenty Four Parganas, Uttar Dinajpur	Darjiling, Haora, Koch Bihar, Kolkata, Murshidabad, North Twenty, Four Parganas, Purba, Medinipur		Bankura, Birbhum, Haora, Hugli, Jalpaiguri, Maldah, Nadia, Puruliya	Barddhaman, Dakshin Dinajpur, Darjiling, Kolkata, Murshidabad, North Twenty, Four Parganas, Paschim, Medinipur, Uttar Dinajpur	South Twenty Four Parganas	Bankura Haora Puruliya	Barddhaman, Birbhum, Dakshin Dinajpur, Hugli, Murshidabad, Nadia, North Twenty Four Parganas, Paschim Medinipur, South Twenty Four Parganas, Uttar Dinajpur	Darjiling, Jalpaiguri, Maldah, Purba Medinipur
**CENTRAL ZONE**									

**Madhya Pradesh**	Chhindwara Gwalior	Betul, Bhopal, Chhatarpur, Hoshangabad, Jabalpur, Khandwa (East Nimar), Satna, Seoni, Shivpuri, Sidhi	Bhind, Damoh, Katni, Narsimhapur, Sheopur, Vidisha	Ahmadnagar, Solapur	Betul, Bhopal, Sagar, Sidhi	Balaghat, Chhindwara, Dindori, Guna, Narsimhapur, Raisen, Sehore, Tikamgarh, Ujjain		Jabalpur,	Chhatarpur, Chhindwara, Indore, Narsimhapur, Shajapur
**NORTH-EAST ZONE**									
**Assam**					Darrang, Kamrup, Metropolitan, Nalbari, Udalguri	Bongaigaon, Dhubri, Golaghat, Sonitpur		Darrang Sivasagar	Barpeta, Cachar, Dibrugarh, Kamrup, Metropolitan, Karimganj, Sonitpur, Tinsukia
**Sikkim**									East District
**Tripura**				North Tripura	South Tripura	West Tripura		Dhalai	

*The endemicity categorization of PPR is based on the scale of the cumulative number of outbreaks, that occurred in the district area and classified into the different endemic level as sporadic (1 outbreak), endemic (low and high endemic districts = 2 to 4 outbreaks), and hyperendemic (> 4 outbreaks) districts

Figure S1. State-wise case fatality rate of PPR in sheep and goats in different zones of the country (1995-2019)

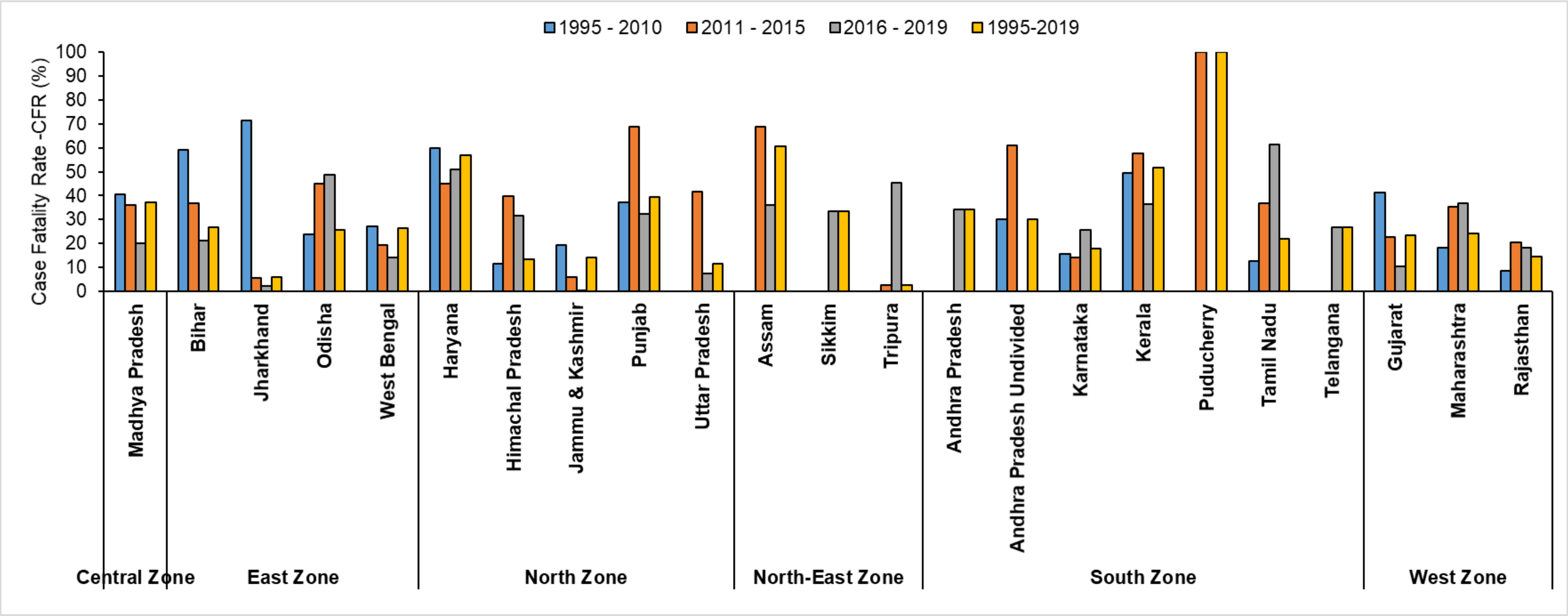

Supplement: Supplementary file 1 [file viruses-13-00480-s001.pdf]
